# Resveratrol Sensitizes Carfilzomib-Induced Apoptosis via Promoting Oxidative Stress in Multiple Myeloma Cells

**DOI:** 10.3389/fphar.2018.00334

**Published:** 2018-05-14

**Authors:** Qian Li, Yuanfang Yue, Lin Chen, Chang Xu, Yan Wang, Liqing Du, Xiaolei Xue, Qiang Liu, Yafei Wang, Feiyue Fan

**Affiliations:** ^1^Tianjin Key Laboratory of Radiation Medicine and Molecular Nuclear Medicine, Institute of Radiation Medicine, Chinese Academy of Medical Sciences and Peking Union Medical College, Tianjin, China; ^2^Tianjin Key Laboratory of Cancer Prevention and Therapy, Tianjin's Clinical Research Center for Cancer, Department of Hematology and Blood and Marrow Transplantation, Tianjin Medical University Cancer Institute and Hospital, National Clinical Research Center for Cancer, Tianjin, China; ^3^Baokang Hospital, Tianjin University of Traditional Chinese Medicine, Tianjin, China; ^4^Institute of Laboratory Animal Sciences, Chinese Academy of Medical Sciences and Peking Union Medical College, Beijing, China

**Keywords:** resveratrol (RSV), carfilzomib (CFZ), proteasome, oxidative stress, autophagy

## Abstract

The proteasome inhibitor is a target therapy for multiple myeloma (MM) patients, which has increased the overall survival rate of multiple myeloma in clinic. However, relapse and toxicity are major challenges for almost all MM patients. Thus, there is an urgent need for an effective and less toxic combination therapy. Here, we demonstrated that a natural compound, resveratrol (RSV) displayed anti-proliferative activity in a dose- and time-dependent manner in a panel of MM cell lines. More importantly, a low concentration of RSV was synergistic with a low dose of the proteasome inhibitor carfilzomib (CFZ) to induce apoptosis in myeloma cells. Further studies showed that mitochondria was a key regulatory site after RSV/CFZ combination treatment. RSV induced the release of second mitochondria-derived activator of caspase (Smac) in a dose-dependent manner and kept the Smac in a high level after combination with CFZ. Also, RSV was additive with CFZ to increase reactive oxygen species (ROS) production. Moreover, a stress sensor SIRT1, with deacetylase enzyme activity, was remarkably downregulated after RSV/CFZ combination, thereby significantly decreasing its target protein, survivin in MM cells. Simultaneously, autophagy was invoked after RSV/CFZ combination treatment in myeloma cells. Further inhibition of autophagy could increase more ROS production and apoptosis, indicating a close linkage between autophagy and proteasome to modulate the oxidative stress. Together, these findings suggest that induction of multiple stress responses after RSV/CFZ combination is a major mechanism to synergistically inhibit MM cell growth and reduce the toxicity of CFZ in MM cells. This study also provides an important rationale for the clinic to consider an autophagy inhibitor for the combination therapy in MM patients.

## Introduction

Multiple myeloma (MM) is a heterogeneous B-cell malignancy characterized primarily by the accumulation of clonal plasma cells in the bone marrow (Anderson et al., 2016), which results in substantial immunosuppression and end-organ damage, including direct and indirect effects on the blood, skeleton, and kidneys (Rajkumar et al., 2011). Although new therapeutic options for MM have improved response rate and increased overall survival rate, most patients with MM will ultimately relapse (Kumar et al., 2008). There is thus an urgent need for more efficient treatment or effective combination to improve the outcome of MM (Dimopoulos et al., 2010).

The proteasome has been validated as an effective target for the treatment of MM (Ocio et al., 2014). Bortezomib is the first generation proteasome inhibitor which demonstrates striking therapeutic effects on relapsed MM (Richardson et al., 2005; Ocio et al., 2014). Following that, carfilzomib (CFZ) is FDA-approved proteasome inhibitor for the treatment of MM patients who are refractory to bortezomib therapy (Vij et al., 2012). As a monotherapy, this drug shows higher overall response rate than bortezomib in clinical trials and about 20% bortezomib refractory patients get benefit from CFZ treatment (Siegel et al., 2012; Vij et al., 2012; Avet-Loiseau et al., 2016). Despite of less side effects when it is compared with bortezomib, CFZ at high dose is susceptible for the cardiac and pulmonary toxicity (Dimopoulos et al., 2015). Therefore, new complementary therapeutic strategies are needed to improve the efficacy and reduce the toxicity of CFZ. Plant polyphenols such as genistein, curcumin, resveratrol, and green tea polyphenols have been implicated to be able to selectively inhibit proteasomes and induce apoptotic cell death *in vitro* and *in vivo* (Landis-Piwowar et al., 2006; Soave et al., 2017). Thus, it is necessary to explore whether these natural polyphenols can be synergistic with CFZ to improve therapeutic effects on MM.

Resveratrol (RSV), a plant-derived polyphenol (trans-3,4′,5-trihydroxystilbene), is found in grapes and other food products. It is one of the most effective and well documented natural compounds with chemo-sensitizing properties and antitumor activities (Jang et al., 1997; Landis-Piwowar et al., 2006). Compelling reports have shown that RSV has a potential to suppress proliferation and induce apoptosis of several types of cancers including solid and hematological tumors (Jang et al., 1997; Ulrich et al., 2006; Bhardwaj et al., 2007; Catalgol et al., 2012; Frazzi et al., 2013). Additionally, RSV displays antioxidant, anti-inflammatory, anti-proliferative, and anti-angiogenic effects on a variety of dieses including cardiovascular diseases, cancer, neurodegenerative diseases (Catalgol et al., 2012). Mitochondria is an important target site for RSV to induce apoptosis (Sareen et al., 2007; van Ginkel et al., 2007). In agreement with this, RSV treatment will give benefit for many disorders, particularly in diseases where oxidative stress plays an important role (Catalgol et al., 2012). Moreover, SIRT1, a NAD+-dependent deacetylase, is regulated by RSV (Knutson and Leeuwenburgh, 2008; Wang et al., 2008). It plays an important role in maintenance the homeostasis of epigenetic gene expression through an acetylation/deacetylation mechanism to modulate the function of many stress-responsive transcription factors, such as p53 and FOXO (Brunet et al., 2004; Motta et al., 2004; Zhang et al., 2011). Importantly, survivin is a SIRT1 target protein which plays a critical role in modulation of apoptosis (Altieri, 2008; Luo and Altieri, 2008). Nevertheless, it needs to be elucidated the mechanism of inhibitory effects on MM cells after RSV/CFZ combination treatment.

We sought here to investigate whether low dose of RSV can sensitize myeloma cells to CFZ-mediated antitumor effects and further understand the underlying mechanisms. Our results demonstrated that RSV and CFZ are synergistic to induce apoptosis in MM cells. An important mechanistic change is that the function of mitochondria is significantly impaired to release ROS production and Smac after RSV/CFZ combination treatment. Furthermore, SIRT1/survivin axis is remarkably attenuated by these two compounds combination. Of note, autophagy is found to be involved in the protection MM cells from oxidative stress and associated apoptosis after RSV/CZF combination treatment. These results suggested that proteasome, autophagy, and mitochondria are closely linked in the modulation of cellular metabolism, stress, and apoptosis. Taken together, RSV/CFZ combination may improve CFZ therapeutic effects with less side effects for human MM patients.

## Materials and methods

### Reagents and antibodies

Carfilzomib (CFZ) was purchased from Onyx Pharmaceuticals (San Francisco, CA, USA). Resveratrol (RSV), N-Acetylcysteine (NAC), methyl-thiazolyl tetrazolium (MTT), 2′, 7′-dichlorofluorescein diacetate (DCFH-DA) fluorescent probe, and dimethyl sulfoxide (DMSO) were from Sigma-Aldrich (St. Louis, MO, USA). 3-methyladenine (3-MA) was obtained from Abmole inhibitor leader (Houston, TX, USA). The CFZ and NAC were dissolved in double-distilled water, and the RSV and 3-MA were dissolved in DMSO, respectively. Primary antibodies of SIRT1 (Cat. 2310), Smac (Cat.15107), survivin (Cat.2808), p-p38 (Cat.4511), p53 (Cat.2527), PARP (Cat.9542), caspase 3 (Cat.9662), LC3-I/II (Cat. 3868), and tubulin (Cat. 2144) were purchased from Cell Signaling Technology (Beverly, MA, USA). Antibody against Tubulin was from Wuhan Boster Biological Technology (Wuhan, Hubei, China). Scrambled siRNA AND SMARTpool: ON-TARGETplus DIABLO (Smac) siRNA were purchased from Dharmacon.

### Cell culture

Human MM cell lines (LP-1, U266, MM.1S, and MM.1R) were purchased from Cell Resource Center of Shanghai Institutes for Biological Sciences (Shanghai, China). Cells were maintained in RPMI-1640 medium (Invitrogen, Frederick, MD, USA) containing 10% fetal bovine serum.

### MTT (3-(4,5-dimethylthiazol-2-YL)-2,5-diphenyltetrazolium bromide) assay

MM cell lines (LP-1, U266, MM.1S, MM.1R) were seeded in 96-well plates at a density of 4 × 10^5^ cells per well. After treatment with different compounds, MTT (final concentration: 0.5 mg/ml) was added into wells. Next, cells were incubated at 37°C for 4 h. The plates were then centrifuged at 300 × g for 10 min at room temperature. Supernatants were removed and DMSO was added to dissolve the MTT formazan crystals. Absorbance was measured at 492 nm using a microplate reader (ELx800; BioTek Instruments, Inc., USA). Growth inhibition was calculated as follows: growth inhibition rate (%) = [(A _control_−A _controlblank_)−(A _sample_−A _sampleblank_)]/(A _control_−A _controlblank_).

### Annexin V analysis of apoptosis

A FITC Annexin V Detection Kit I (Keygene, KGA108) was used to quantify apoptosis of MM cell lines through flow cytometry according to the manufacturer's instructions. In brief, MM cells were seeded in 10-cm dishes. The next day, the cells were treated with different compounds as indicated. Cells were suspended in 1 × binding buffer and 1 × 10^5^ cells were stained simultaneously with FITC-labeled annexin V (FL1-H) and propidium iodide (PI) (FL2-H). The cells were analyzed using a BD Accuri™ C6 plus flow cytometer (Becton Dickinson). Cell populations were categorized using the following criteria: Q1: necrotic cells (Annexin V-negative/PI-positive), Q2: late apoptotic cells (Annexin V/PI-double positive), Q3: live cells (Annexin V/PI-double negative), and Q4: early apoptotic cells (Annexin V-positive/PI-negative). Apoptotic rate was determined as the percentage of Q2+Q4.

### Cell cycles analysis

MM cells were plated into 60-mm plates at a density of 1 × 10^6^ cells. After 24 h, cells were treated with different compounds as indicated. Then, cells were harvested and gradually fixed with 70% EtOH on ice. After staining with propidium iodide (PI), cells were analyzed using a FACSort flow cytometer (Becton Dickinson Accuri C6).

### Intracellular ROS production

MM Cells were pretreated with or without NAC (3 mmol/L) or 3-MA (1 mmol/L) for 2 h. Then, cells were treated with various compounds for indicated times. Cells were harvested and washed with PBS. Next, cells were suspended in RPMI-1640 medium containing 0.1 μmol/L DCFH-DA fluorescent dye for 30 min at 37°C. Fluorescence intensity was detected through a flow cytometer (Becton Dickinson).

### Western blotting analysis

After different treatment, cells were washed with ice-cold PBS. Cell lysates were harvested in RIPA lysis buffer (Beijing Kangwei-century Biological Technology, Beijing, China). Protein concentration was determined using the Bio-Rad protein assay. Equal amounts of protein per sample were subjected to SDS-PAGE gel electrophoresis. Then, protein was transferred to PVDF membranes (Beijing Dingguo Biological Technology, Beijing, China). After blocking with 5% non-fat milk, the membranes were probed with the primary and secondary antibodies. Signal was developed with the enhanced chemiluminescence method (Beijing CW, Beijing, China), and visualized using the Kodak Image Station.

### Small interfering RNA transfection

MM cells were loaded in a 12-well plate with the density of 1 × 10^6^ cells. Next day, cells were transfected with Smac siRNA and the scrambled control siRNA as manufacturer's suggestion. Culture media were changed after 24 h transfection. Cells were harvested after 72 h transfection for Western blotting.

### Quantitative real-time reverse transcription-PCR

Total RNA isolated from cells using an RNeasy Micro kit (Qiagen) was converted to first-strand cDNA using a high-capacity cDNA reverse transcription kit (Applied Biosystem). Quantitative real-time PCR assays were performed with SYBR Green PCR Master Mix (Applied Biosystems) and a QuantStudio 6 Flex real-time PCR System (Applied Biosystems).

### Statistical analysis

All data were presented as mean ± standard deviation (SD) after three independent experiments. Statistical significance was determined using Student's *t*-test and One-Way ANOVA with *P*-values < 0.05 representing significance.

## Results

### RSV inhibits myeloma cell growth through induction of apoptosis

**Figure 1:**
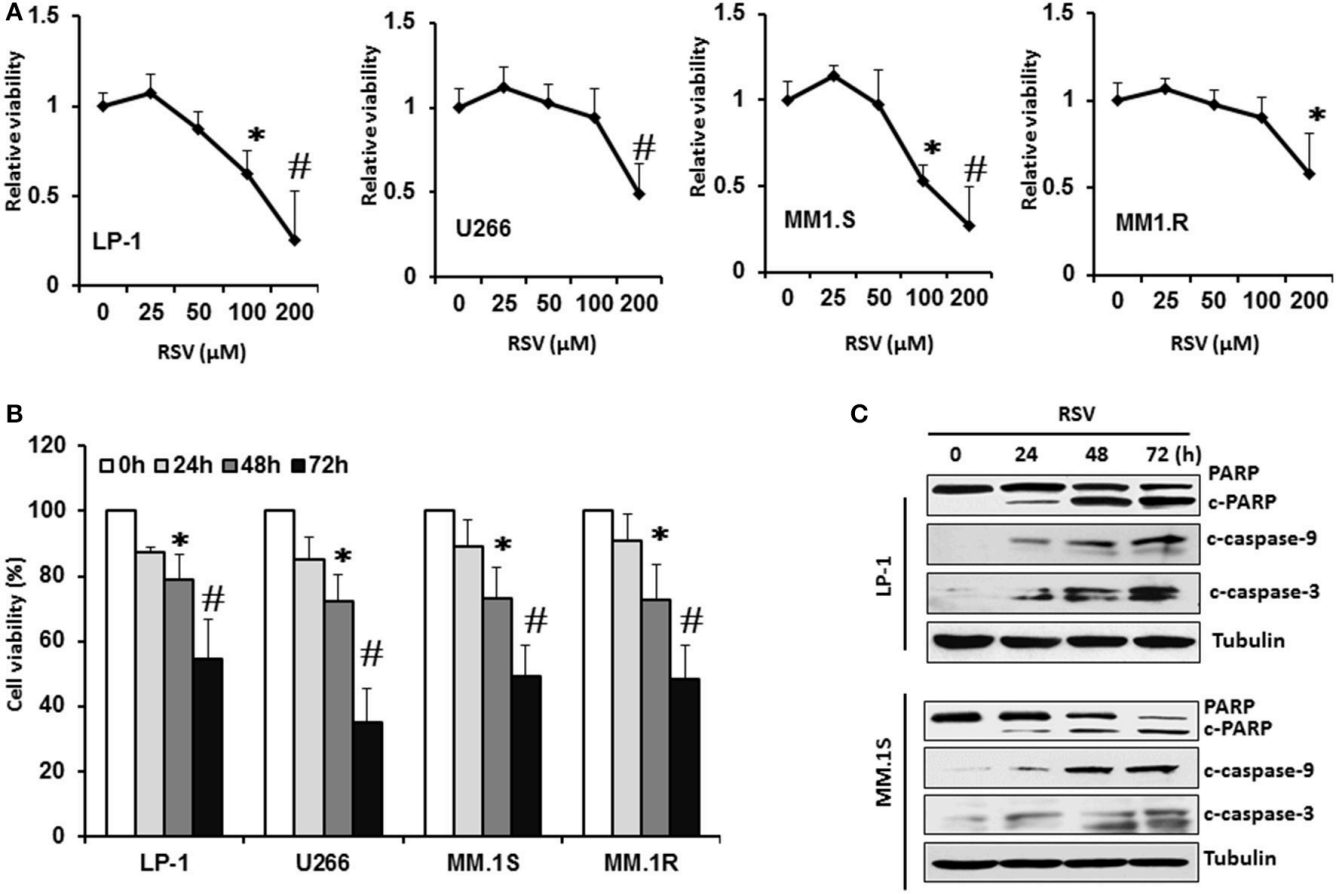
Resveratrol inhibited MM cell growth. **(A)**, LP-1, U266, MM.1S, and MM. 1R cells were treated with vehicle control (0.1% DMSO) and various concentrations (25, 50, 100, 200 μM) of RSV for 24 h. Cell viability was detected by MTT assay. **(B)**, MM cells were treated with the respective IC50 concentration of RSV for 24, 48, and 72 h. Cell viability was detected by MTT assay. *P < 0.05; #*P* < 0.01 compared with the respective control. **(C)**, LP-1 and MM.1S cells were treated with RSV at 50 μM for 24, 48, and 72 h. Cell lysates were harvested. Cleaved PARP and caspase−3/−9 were detected by Western blotting. Tubulin was used as a loading control.

We first investigated the effect of RSV on cell viability in a panel of MM cell lines. Four MM cell lines (LP-1, U266, MM.1S, and MM.1R) were treated with a vehicle control (0.1% DMSO) and various concentrations (25, 50, 100, 200 μM) of RSV for 24 h. Cell viability was measured by MTT assay. As shown in Figure 1A, RSV remarkably inhibited MM cell viability in a dose-dependent manner in four MM cell lines. LP-1 and MM.1S were relatively sensitive to the RSV treatment (Figure 1A). All of these MM cells were then treated with RSV at their individual IC50 for 24, 48, and 72 h to detect inhibitory effects through MTT assay. RSV started to inhibit cell viability after 48 h treatment in four cell lines. This inhibitory effects were increased further after 72 h exposure to RSV (*P* < 0.01; Figure 1B). Consistently, apoptotic biomarkers cleaved PARP and caspase−3/−9 were observed after 24 h treatment with RSV in LP-1 and MM.1S (Figure 1C). The cleaved PARP and caspase−3/−9 were increased more when the treatment time was prolonged to 48 and 72 h (Figure 1C and Supplementary Figure 1). These results demonstrated that RSV can inhibit MM cell viability and induce apoptosis.

### RSV sensitizes CFZ-induced apoptosis in MM cell lines

**Figure 2:**
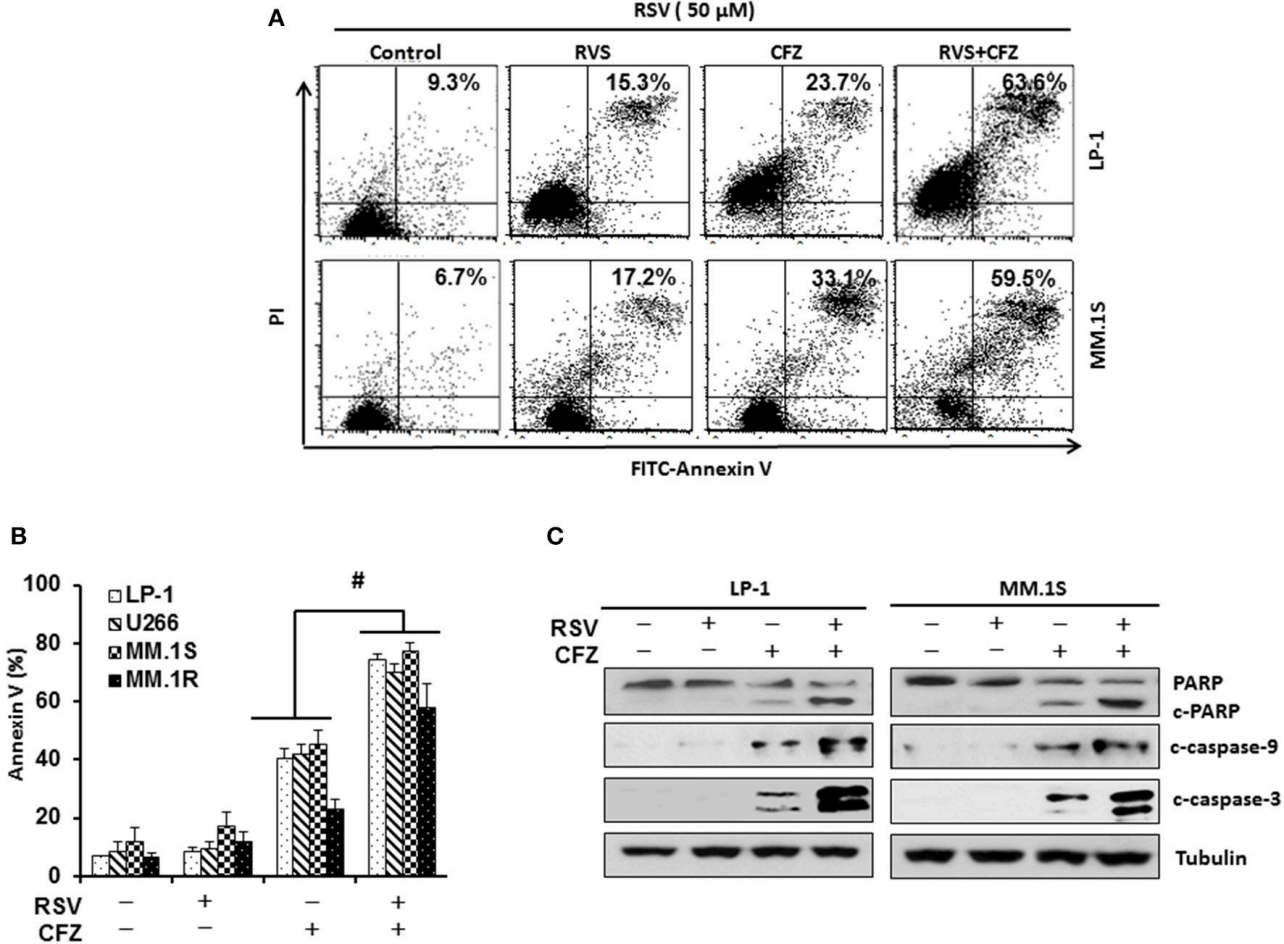
Resveratrol sensitized MM cells to CFZ-induced apoptosis. **(A)**, LP-1 and MM.1S cells were treated with RSV (50 μM), CFZ (40 nM), or in a combination of them for 24 h. FITC-Annexin V staining was detected through flow cytometry. **(B)**, Cells of four MM cell lines (LP-1, U266, MM.1S, and MM.1R) were treated with RSV (50 μM), CFZ (40 nM), or in a combination of them for 24 h. Data represent the mean ± SD for three separate experiments performed in triplicate. #*P* < 0.01, CFZ+RSV groups vs single agent treated groups as indicated. **(C)**, LP-1 and MM.1S cells were treated with RSV (50 μM), CFZ (40 nM) or their combination for 24 h. Cleaved PARP and caspase−3/−9 were detected by Western blotting. Tubulin was used as a loading control.

To examine the impact of RSV on the sensitivity of carfilzomib (CFZ) to induce apoptosis after combination treatment, LP-1 and MM.1S were treated with CFZ (40 nM), RSV (50 μM), or in a combination for 24 h. Annexin V binding assay was performed to detect apoptosis through flow cytometry. Our results showed that both RSV and CFZ increased apoptosis in LP-1 and MM.1S cells. Importantly, they synergistically increased apoptosis when they were administered together (Figure 2A). Then, we treated other two cell lines with compound alone or in a combination. Consistently, CFZ was remarkably stronger than RSV to induce apoptosis. Combination of RSV and CFZ further increased the cleavage of PARP and caspase−3/−9 as compared to that in cells treated with either RSV or CFZ alone (*P* < 0.01) (Figure 2B). In line with this result, combination RSV and CFZ increased more cleaved PARP and caspase−3/−9 than that in single agent treated groups (Figure 2C and Supplementary Figure 2). All of these results suggested that RSV sensitizes myeloma cells to CFZ-induced apoptosis.

### RSV and CZF differentially modulates cell cycles of MM cell lines

**Figure 3:**
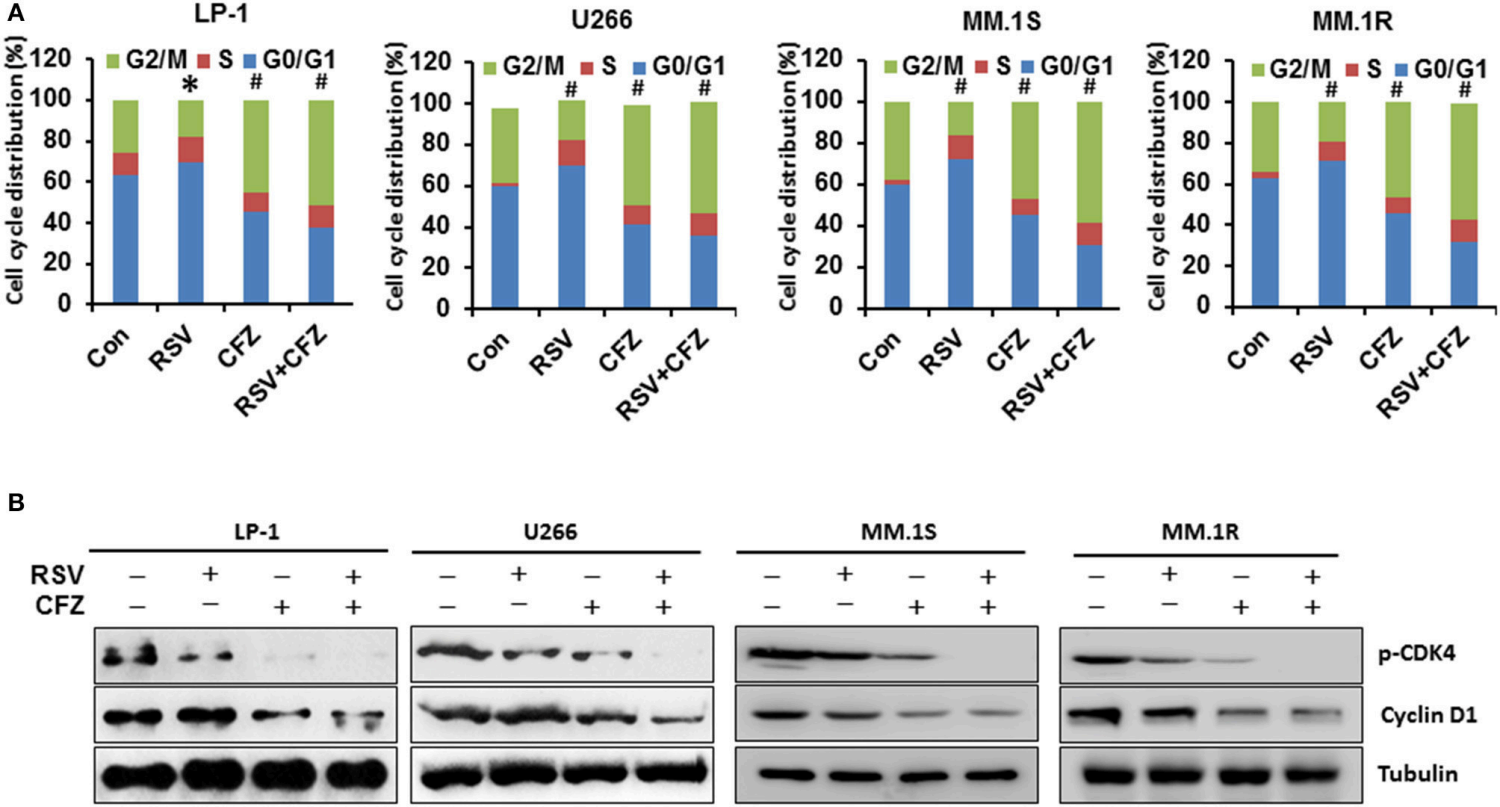
Differential effects on cell cycles by RSV and CFZ in MM cells. **(A)**, Four MM cell lines LP-1, U266, MM.1S, and MM.1R were treated with RSV (50 μM), CFZ (40 nM), or in a combination of them for 24 h. Cells were harvested and gradually fixed with ethanol for cell cycles analysis through flow cytometery. *P < 0.05; #*P* < 0.01 compared with the respective control. **(B)**, Four MM cell lines LP-1, U266, MM.1S, and MM.1R were treated the same as in **(A)**. Cell lysates were harvested. Expression of phosphorylated CDK4 and cyclin D1 was measured by Western blotting. Tubulin was used as a loading control.

To investigate whether RSV and CFZ have any influence on cell cycles to inhibit cell growth, MM cells were treated with RSV, CFZ, or in a combination as described above. Cell cycles were detected by flow cytometry. The result showed that RSV treatment arrested LP-1 cells at G0/G1 phase of cell cycles compared with control (*P* < 0.05), whereas CFZ treatment arrested LP-1 cells at G2/M phase of cell cycles (*P* < 0.01). And RSV/CFZ combination treatment mildly increased the percentage of G2/M compared with CFZ alone treated LP-1 cells (Figure 3A). The similar trends were observed in the U266 cells. RSV significantly arrested U266 cells at G0/G1 phase. RSV/CFZ combination was similar to CFZ alone treatment, which both arrested U266 cells at G2/M phase (*P* < 0.01; Figure 3A). As for another two MM cell lines: MM.1S and MM.1R cells, RSV still arrested cells at G0/G1 phase, while CFZ arrested cells at G2/M phase. And combination treatment had the similar cell cycles as that cells treated with CFZ (Figure 3A). Then, we examined the cell cycles associated signaling pathways CDK4 and cyclinD1. Our results demonstrated that CFZ was more potent than RSV to remarkably reduce phosphorylation of CDK4 and cyclinD1. Combination treatment decreased more phosphorylation of CDK4 and cyclinD1, particularly in phosphorylation of CDK4 (Figure 3B and Supplementary Figure 3). These results indicated that RSV and CFZ have distinct function to regulate cell cycles of MM cell lines. They are not additive to arrest MM cells at certain phase of cell cycles but on signaling pathways associated with cell cycles.

### RSV is additive with CFZ to increase oxidative stress in MM cells

**Figure 4:**
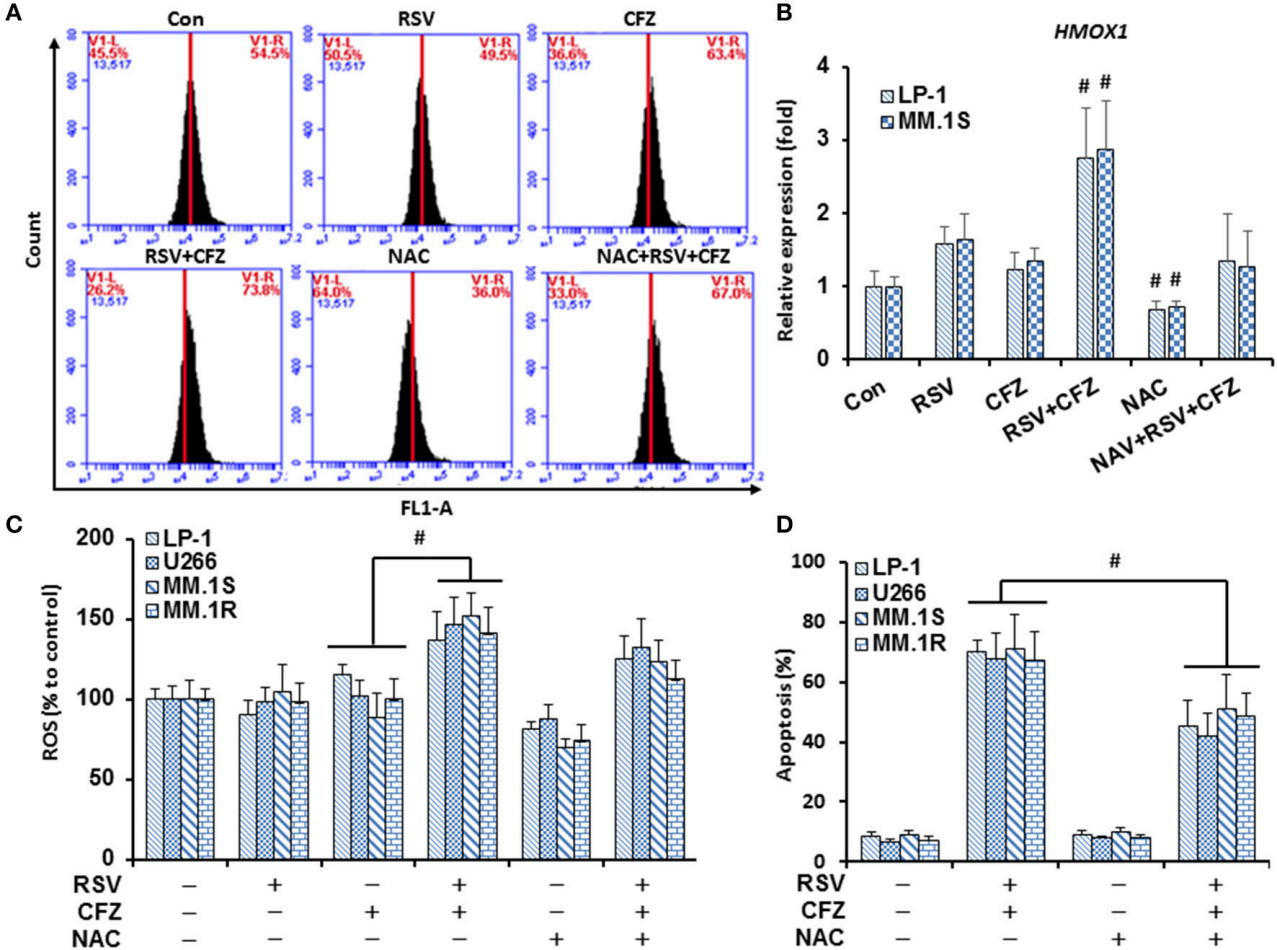
RSV and CFZ combination increased ROS production in MM cells. **(A)**, LP-1 cells were treated with RSV (50 μM), CFZ (40 nM), or their combination treatment with or without pretreatment of NAC (3 mM, 2 h). Then, cells were harvested after 24 h for DCFH-DA staining to measure ROS production by flow cytometry. **(B)**, LP-1 and MM.1S cells were seeded in 6-well plates and were treated the same as in **(A)**. Cells were harvested in Trizol. Expression of HMOX1 mRNA was quantified by RT-PCR. ^#^*P* < 0.01. **(C)**, LP-1, U266, MM.1S, and MM.1R cells were treated the same as in **(A)**. ROS production was measured by flow cytometry. *P* < 0.001, * compared with groups as indicated. **(D)**, LP-1, U266, MM.1S, and MM.1R cells were treated the same as in **(A)**. Next, cells were harvested to evaluate apoptosis through flow cytometry using FITC-Annexin V/PI staining assay. *P* < 0.01, * compared with groups as indicated. Data represent the mean ± SD for three separate experiments performed in triplicate.

Dysfunction of mitochondria is an important mechanism for cells to undergo apoptosis. Reactive oxygen species (ROS) are major products released from mitochondria to cause apoptosis. To make clear whether such mechanisms are responsible for RSV/CFZ-induced apoptosis, MM cells were treated with RSV and CFZ the same as above. Intracellular ROS production was measured using DCFH-DA fluorescent dye through flow cytometery. As shown in Figure 4A, RSV did not increase ROS production, CFZ had a trend to increase ROS in LP-1 cells when they were compared with control cells, but without significant difference. Importantly, combination RSV and CFZ could remarkably increase the ROS production (Figure 4A). The free radical scavenger NAC (3 mM) clearly reduced the ROS levels, but was unable to completely block the ROS induction by the combination RSV and CFZ (*P* = 0.128) (Figure 4A). To confirm the regulation of the oxidative stress by the compounds, an oxidative stress indicator HMOX1 expression was quantified by real-time RT-PCR. Our results showed that RSV had a trend to increase the expression of HMOX1, but without significant difference. CFZ did not increase the HMOX1 expression (*P* < 0.01). However, the combination of two compounds clearly upregulated the levels of HMOX1. NAC could reduce the basal levels of HMOX1 and inhibited the upregulation of HMOX1 after combination treatment (Figure 4B). The similar alteration of ROS production was observed in four MM cell lines (Figure 4C). Further examination of the apoptotic effects on four MM cell lines, combination RSV and CFZ significantly increased apoptosis (*P* < 0.01). NAC did not induce apoptosis. Pre-treatment with NAC in RSV/CFZ combination group could partially block the apoptosis (Figure 4D). These results suggested that induction of oxidative stress is one of the mechanisms to inhibit MM cell growth by RSV/CFZ combination.

### RSV modulates stress-associated pathways after combination with CFZ

**Figure 5:**
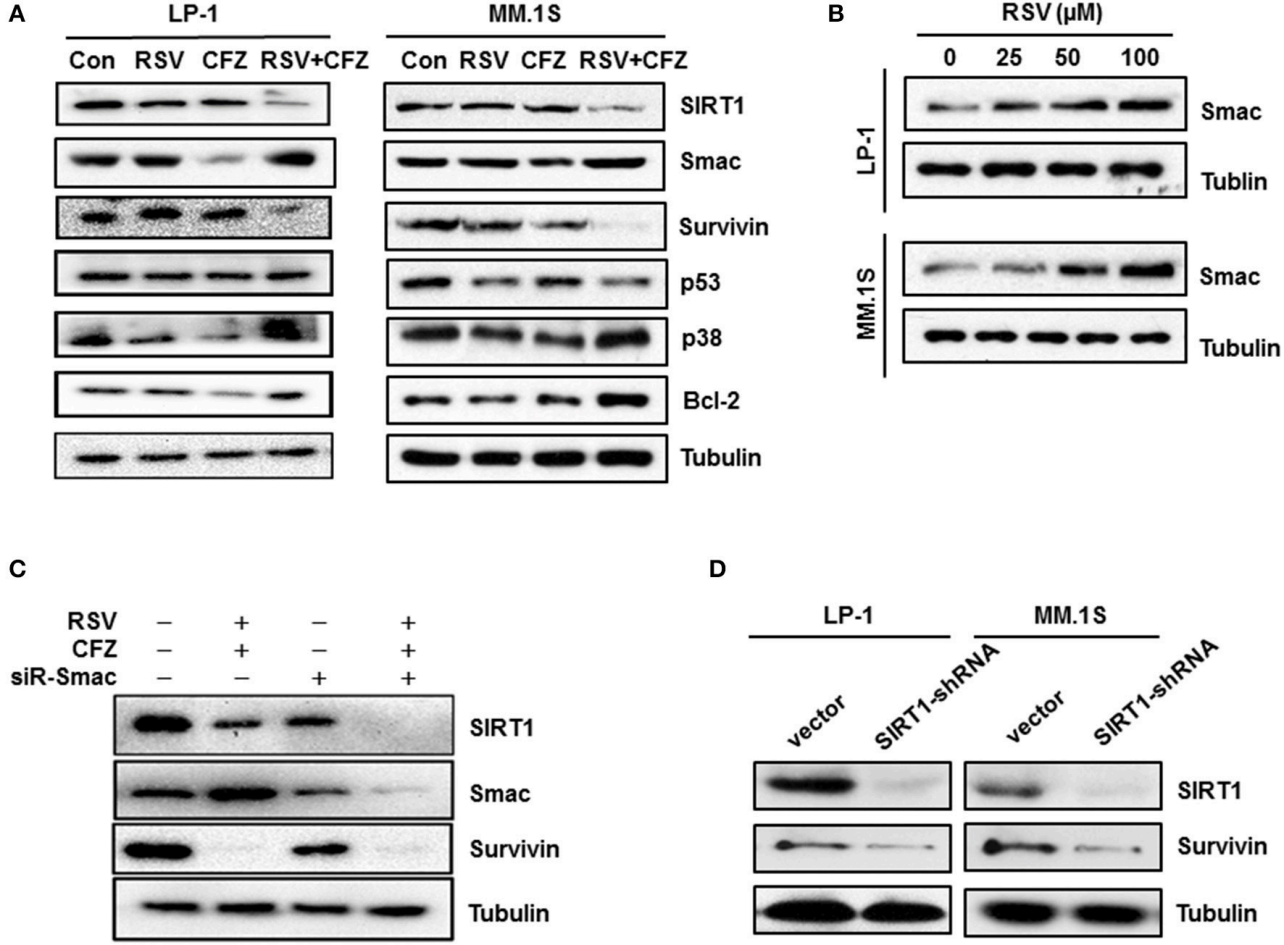
Resveratrol increased stress-associated pathways after combination with CFZ. **(A)**, LP-1 and MM.1S cells were treated with RSV (50 μM), CFZ (40 nM) or a combination of them for 24 h. Then, cell lysates were harvested. SIRT1, Smac, Survivin, p53, and phosphorylated p38 were detected through Western blotting. Tubulin was used as a loading control. **(B)**, LP-1 and MM.1S cells were treated with different concentrations (25, 50, 100, μM) of RSV for 24 h. Smac protein levels were detected through Western blotting. Tubulin was used as a loading control. **(C)**, LP-1 cells were transfected with scrambled siRNA and Smac siRNA for 72 h. Then, cells were treated with vehicle control or RSV (50 μM)/CFZ (40 nM) combination for 24 h. Cells lysates were harvested to detect Smac, SIRT1, and survivin expression through Western blotting. Tubulin was used as a loading control. **(D)**, LP-1 and MM.1S cells were transfected with vector control and SIRT1 specific shRNA for 72 h. Then, cells lysates were harvested to detect SIRT1 and survivin expression through Western blotting. Tubulin was used as a loading control.

To gain further insight into the molecular mechanisms underlying apoptosis sensitivity upon combined RSV/CFZ treatment, LP-1 cell, and MM.1S cells were treated with the compounds the same as above. Individual RSV and CFZ treatment did not significantly alter expression levels of SIRT1, a deacetylase enzyme that regulates the activity of several transcriptional factors and enzymes in response to stress (Strycharz et al., 2018). Interestingly, they were synergistic to reduce SIRT1 in both cell lines (Figure 5A). Another mitochondrial protein (Kamata et al., 2017), Smac expression levels were increased by the RSV treatment, whereas CFZ significantly decreased Smac in LP-1 cells. Combination RSV and CFZ kept the high levels of Smac the same as RSV alone treated cells (Figure 5A and Supplementary Figure 4). RSV increased Smac expression levels in a dose-dependent manner in LP-1 and MM.1S cells (Figure 5B and Supplementary Figure 5A). Similar as in the regulation of SIRT1, RSV/CFZ significantly downregulated survivin (Figure 5A), a key regulator to against apoptosis (Dohi et al., 2004). There was no significant alteration of p53 expression after compounds alone or combination treatment (Figure 5A). Notably, RSV/CFZ combination increased the expression of Bcl-2 (Figure 5A and Supplementary Figure 4). As for the stress-responsive signal p38, RSV, or CFZ alone treatment downregulated phosphorylated p38, while combination of them remarkably increased phosphorylated p38 (Figure 5A and Supplementary Figure 4). Additionally, survivin has been reported to bind to Smac in the mitochondria to regulate apoptosis (Sun et al., 2005). To further investigate the relationship between these mitochondria-associated proteins, Smac was knocked down by a specific small interfering RNAs (siRNA). Protein levels of Smac were effectively downregulated (Figure 5C and Supplementary Figure 5B). SIRT1 and survivin were downregulated when the Smac was knocked down (Figure 5C). However, depletion of Smac couldn't rescue the remarkable downregulation of SIRT1 and survivin by RSV/CFZ combination treatment (Figure 5C). Then, downregulation of SIRT1 by specific shRNA, the protein levels of survivin were decreased (Figure 5D and Supplementary Figures 5C,D), indicating survivin is the downstream signal of SIRT1 in MM cells.

### Combined RSV and CFZ treatment induces protective autophagy in MM cells

**Figure 6:**
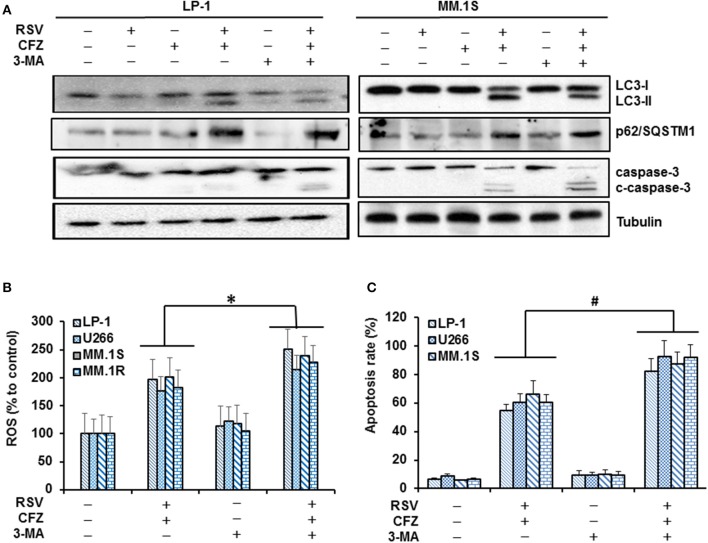
Autophagy was involved in the modulation of oxidative stress after RSV/CFZ combination treatment. **(A)**, LP-1 and MM.1S cells were treated with RSV (50 uM), CFZ (40 nM) or the combination of RSV and CFZ with or without pre-treatment 3-MA (1 mM for 2 h). Then, cell lysates were harvested after 24 h treatment. LC3-I/II, p62/SQSTM1, and caspase-3/cleaved-caspase-3 were detected by Western blotting. **(B)**, LP-1, U266, MM.1S, and MM.1R cells were treated the same as in **(A)**. Cells were harvested to measure ROS production through flow cytometery. *P < 0.05, compared with indicated group. **(C)**, LP-1, U266, MM.1S, and MM.1R cells were treated the same as in **(A)**. Then, cells were harvested to measure apoptosis by flow cytometry using FITC-Annexin V/PI staining assay. #*P* < 0.001, compared with indicated group. Data represent the mean ± SD for three separate experiments performed in triplicate.

Autophagy is closely linked with proteasome and functions as an indispensable metabolic way to maintain homeostasis and promote cell survival (Madeo et al., 2015). Many chemotherapeutic drugs can induce autophagy in tumor cells, but the function of autophagy as a pro-survival or pro-apoptotic signal relies on complicated cellular circumstance (Sui et al., 2013). Thus, we investigated whether RSV/CFZ combination treatment could induce autophagy in MM cells. Our results showed that RSV or CFZ alone treatment did not increase LC3-II and p62/SQSTM1, two biomarkers of autophagy. However, RSV/CFZ combination increased levels of LC3-II and p62/SQSTM1. Higher levels of LC3-II could be partially blocked by the autophagy inhibitor 3-MA, but not for p62/SQSTM1 (Figure 6A). To examine whether 3-MA can regulate apoptosis or not, caspase-3 cleavage was used as a biomarker to evaluate apoptosis. Similar as results shown above, combined RSV/CFZ increased more cleaved caspase-3 than compounds alone. Adding into MA-3 clearly further increased cleaved caspase-3 in LP-1 and MM.1S cells, indicating that blocking of autophagy enhanced CFZ/RSV induced apoptosis in MM cells (Figure 6A and Supplementary Figure 6). To further evaluate whether ROS generation is related with autophagy, four MM cells were treated with combination of CFZ and RSV in the presence or absence of 3-MA. Our results demonstrated that RSV/CFZ significantly increased the ROS production. 3-MA itself did not alter the levels of ROS. Importantly, 3-MA increased more ROS production in RSV/CFZ combination treated MM cells (*P* < 0.05; Figure 6B). In line with the ROS production, pre-treatment with 3-MA significantly increased apoptosis of MM cells induced by CFZ and RSV (*P* < 0.01; Figure 6C). These results suggested that autophagy induced by RSV/CFZ combination treatment is involved in the regulation of oxidative stress.

## Discussion

The proteasome inhibitors (such as bortezomib and carfilzomib) have been confirmed as effective target therapy for multiple myeloma. However, severe toxicity and drug resistance are major challenges for these compounds. In the present study, we demonstrate that low dose of a natural compound, resveratrol (RSV) sensitizes myeloma cells to CFZ-induced apoptosis. An important mechanism is that RSV/CFZ combination creates a stressful microenvironment in MM cells and triggers many stress-associated responses, including increasing oxidative stress and decreasing SIRT1/survivin axis. Simultaneously, autophagy is invoked to protect cells from RSV/CFZ-induced oxidative stress. Based on our results, RSV sensitizes CFZ to release more ROS production and increase oxidative stress indicator HMOX1, which indicates that mitochondria is a major organelle targeted by the RSV/CFZ combination treatment. Therefore, we focused on the energy stress sensor SIRT1/survivin and mitochondria associated protein Smac in the current study.

It is well known that proteasome is the primary target for CFZ to treat myeloma patients (Richardson et al., 2005; Ocio et al., 2014). RSV also has a potential to inhibit the function of proteasome (Dimopoulos et al., 2015). However, our results demonstrate that the mitochondria is a critical regulatory site that is remarkably impaired after RSV/CFZ combination treatment, thereby synergizing CFZ-induced apoptosis. More evidence indicates that induction of oxidative stress has been considered as an important mechanism for the proteasome inhibitors to induce apoptosis in MM cells (Nerini-Molteni et al., 2008; Fink et al., 2016). In line with these observations, our results demonstrate that low doses of RSV/CFZ combination increase more of ROS production. Additionally, the pro-apoptotic protein, Smac is released from the mitochondria in a dose-dependent manner after RSV treatment, which has the function to antagonize inhibitors of apoptosis proteins (IAPs) (Verhagen et al., 2000). The Smac mimetic has synergistic effects with chemo- and immunotherapy to anticancer (Kamata et al., 2017; Kim et al., 2017). Consistently, the Smac mimetic has been observed to improve bortezomib therapeutic effects in B-cell non-Hodgkin lymphoma cells (Bhatti et al., 2017). In our study, CFZ doesn't increase the Smac in MM cells, but combination with RSV keeps the Smac in a high level. This finding suggests that upregulation of Smac by RSV is one of the mechanisms to increase apoptosis after combination treatment with CFZ in MM cells.

Survivin, a member of the family of IAPs, is a key regulator to defend apoptosis (Mita et al., 2007). Functionally, survivin binds to the X-linked inhibitor of apoptosis protein (XIAP) and then enhances XIAP stability against ubiquitin-dependent degradation (Dohi et al., 2007). Emerging evidence suggests that survivin can bind to Smac in mitochondria and interacts with Smac to regulate apoptosis (Song et al., 2003; Ceballos-Cancino et al., 2007). To investigate whether high levels of Smac are responsible for the suppression of survivin, Smac was knocked down by a specific siRNA. However, our results show that depletion of Smac cannot rescue the dramatically decreasing of survivin by the RSV/CFZ combination treatment, indicating other mechanisms are involved. Notably, survivin is found to be directly modulated by a stress sensor SIRT1 via binding to its promoter or epigenetic chromatin modifications (Luo and Altieri, 2008; Han et al., 2017). In accordance with the alteration of survivin, SIRT1 is also significantly downregulated by the RSV/CFZ combination treatment. The mechanisms remain unclear yet. More reports demonstrate that SIRT1 is a putative target of RSV in human tissues and solid tumor models (Knutson and Leeuwenburgh, 2008; Wang et al., 2008; Frazzi et al., 2013). Nevertheless, our results show that RSV does not remarkably decrease the SIRT1/survivin axis in MM cell lines. A recent observation indicates that ROS accumulation in tumor cells can trigger down-regulation of SIRT1 to activate apoptosis (Yang et al., 2012). It needs to make a note here that many Bcl-2 family members might be regulated by the combination treatment although mechanisms remain unclear. Anti-apoptotic protein Bcl-2 is increased after RSV/CFZ combination treatment. This is in line with other group's finding that CFZ sensitizes rituximab-resistant lymphoma cells to chemotherapy but upregulates Bcl-2 expression (Gu et al., 2013), which implies a complex modulation among these mitochondria-associated proteins to decide cell fate. All of these findings demonstrate that these stress-associated signals are cross talking with each other to integrally modulate the apoptosis.

Finally, autophagy and proteasome are functionally coupled that leads to the degradation of damaged organelles and proteins within lysosomes to mitigate metabolic stress (Wojcik, 2013), which have recently gained much more attention for its paradoxical relationship with apoptosis. Autophagy can either prevent apoptosis or enhance apoptosis depending on the cellular context (Tsujimoto and Shimizu, 2005; Degenhardt et al., 2006; Zhang et al., 2014). In the current study, RSV/CFZ combination treatment induces protective autophagy, a defense mechanism to protect cells from undergoing oxidative stress-induced apoptosis. Although mitochondria-associated stress plays a critical role in the apoptosis after RSV/CFZ combination treatment, it is needed to mention that excessive amounts of protein produced by myeloma cells will accumulate in the endoplasmic reticulum and cause stress (Sun et al., 2017). Subsequently, many stress-associated signaling pathways related with metabolism, inflammation, and apoptosis are activated. It is under investigation how these cellular organelles interact with each other in the settings of stress induced by two compounds.

## Conclusions

Many stress responses related with mitochondria, proteasome, and autophagy are induced by the RSV/CFZ combination in MM cells, which are major mechanisms thereby synergistically inhibiting MM cell growth. All of these findings provide an important rationale for the future exploration of targeting the stress response with novel compounds to improve the therapeutic effects of the proteasome inhibitor on myeloma patients.

## Author contributions

YW, FF, and QLiu: contributed to the conception and design of the research, completed the experimental work, prepared for the initial manuscript draft; LC, CX, YW, LD, and XX: performed experiments and analyzed data; QLi, YW, and FF: contributed to the conception and design of the research and manuscript development, editing, and revisions. All authors have read and approved the final manuscript.

### Conflict of interest statement

The authors declare that the research was conducted in the absence of any commercial or financial relationships that could be construed as a potential conflict of interest.
