# Supplementary material for: Resveratrol Sensitizes Carfilzomib-Induced Apoptosis via Promoting Oxidative Stress in Multiple Myeloma Cells
Source: Front Pharmacol. 2018 May 14;9:334. doi: 10.3389/fphar.2018.00334 (PMC5961230; doi:10.3389/fphar.2018.00334)
Supplement: Supplementary file 1 [file DataSheet_1.docx]

**Supplementary Figure Legends**

Supplementary Figure S1. Quantification of Western blot results in Figure 1C with Image J software.

Supplementary Figure S2. Quantification of Western blot results in Figure 2C with Image J software.

Supplementary Figure S3. Quantification of Western blot results in Figure 3B with Image J software.

Supplementary Figure S4. Quantification of Western blot results in Figure 5A with Image J software.

Supplementary Figure S5. Quantification of Western blot results in Figure 5B-D with Image J software.

Supplementary Figure S6. Quantification of Western blot results in Figure 6A with Image J software.
