# Supplementary material for: Resveratrol Sensitizes Carfilzomib-Induced Apoptosis via Promoting Oxidative Stress in Multiple Myeloma Cells
Source: Front Pharmacol. 2018 May 14;9:334. doi: 10.3389/fphar.2018.00334 (PMC5961230; doi:10.3389/fphar.2018.00334)
Supplement: Supplementary file 2 [file Presentation_1.PPTX]

## Slide 1
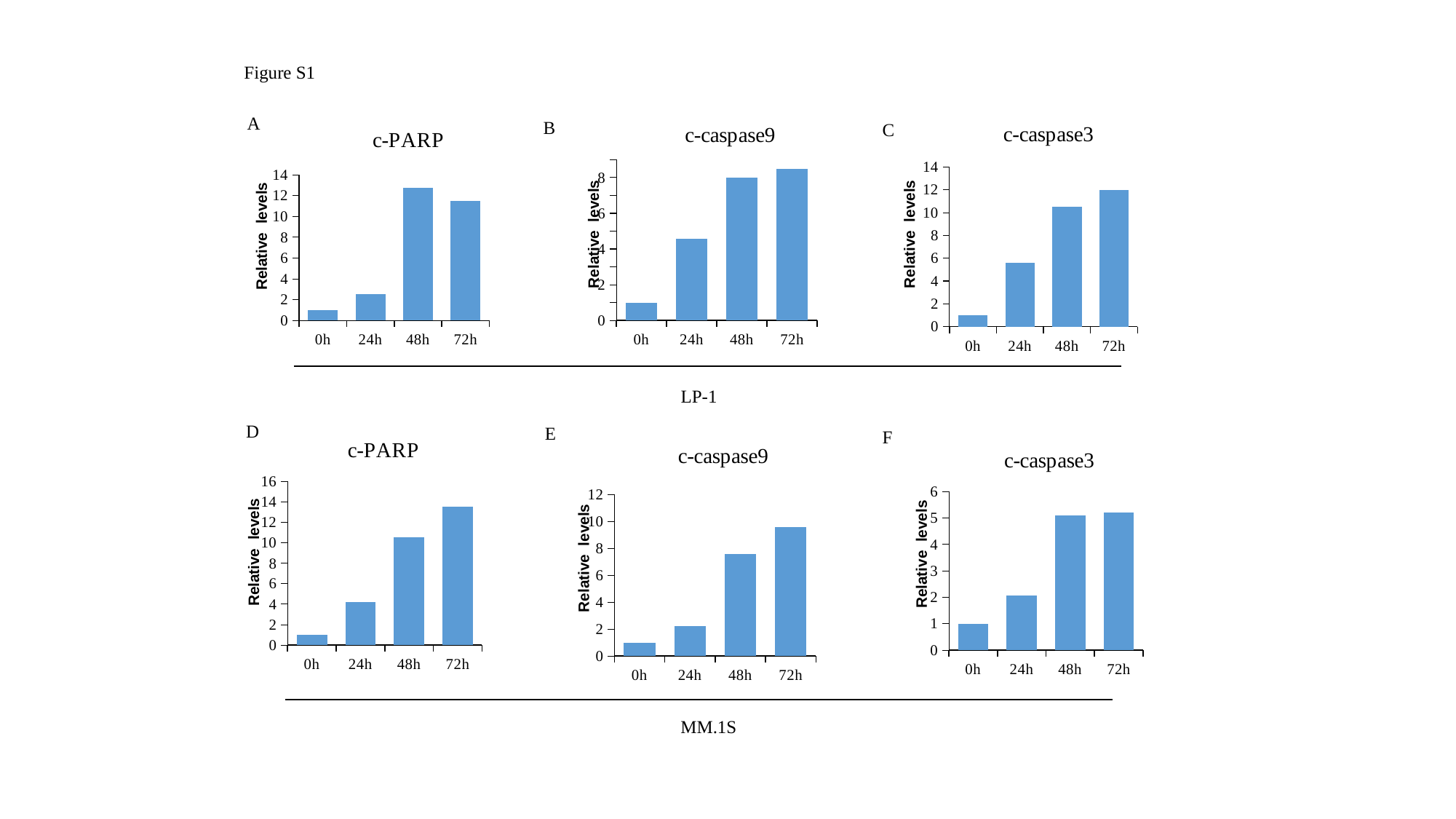

Figure S1
### Chart: c-caspase3
| Category | |
|---|---|
| 0h | 1.0 |
| 24h | 5.621767636433239 |
| 48h | 10.49042122188307 |
| 72h | 12.0 |Relative levels
A
### Chart: c-caspase9
| Category | |
|---|---|
| 0h | 1.0 |
| 24h | 4.555350444949563 |
| 48h | 8.016800943635669 |
| 72h | 8.5 |Relative levels
B
C
### Chart: c-PARP
| Category | |
|---|---|
| 0h | 1.0 |
| 24h | 2.5286940866132306 |
| 48h | 12.725685612591164 |
| 72h | 11.491606915269365 |Relative levels
LP-1
D
E
F
### Chart: c-PARP
| Category | |
|---|---|
| 0h | 1.0 |
| 24h | 4.181009166643756 |
| 48h | 10.583181513011237 |
| 72h | 13.513277812851973 |Relative levels
### Chart: c-caspase3
| Category | |
|---|---|
| 0h | 1.0 |
| 24h | 2.0704115541859376 |
| 48h | 5.1006985951889 |
| 72h | 5.221416876702301 |Relative levels
### Chart: c-caspase9
| Category | |
|---|---|
| 0h | 1.0 |
| 24h | 2.20090482337145 |
| 48h | 7.576318922119254 |
| 72h | 9.55579675196185 |Relative levels
MM.1S
