# Supplementary material for: Resveratrol Sensitizes Carfilzomib-Induced Apoptosis via Promoting Oxidative Stress in Multiple Myeloma Cells
Source: Front Pharmacol. 2018 May 14;9:334. doi: 10.3389/fphar.2018.00334 (PMC5961230; doi:10.3389/fphar.2018.00334)
Supplement: Supplementary file 3 [file Presentation_2.PPTX]

## Slide 1
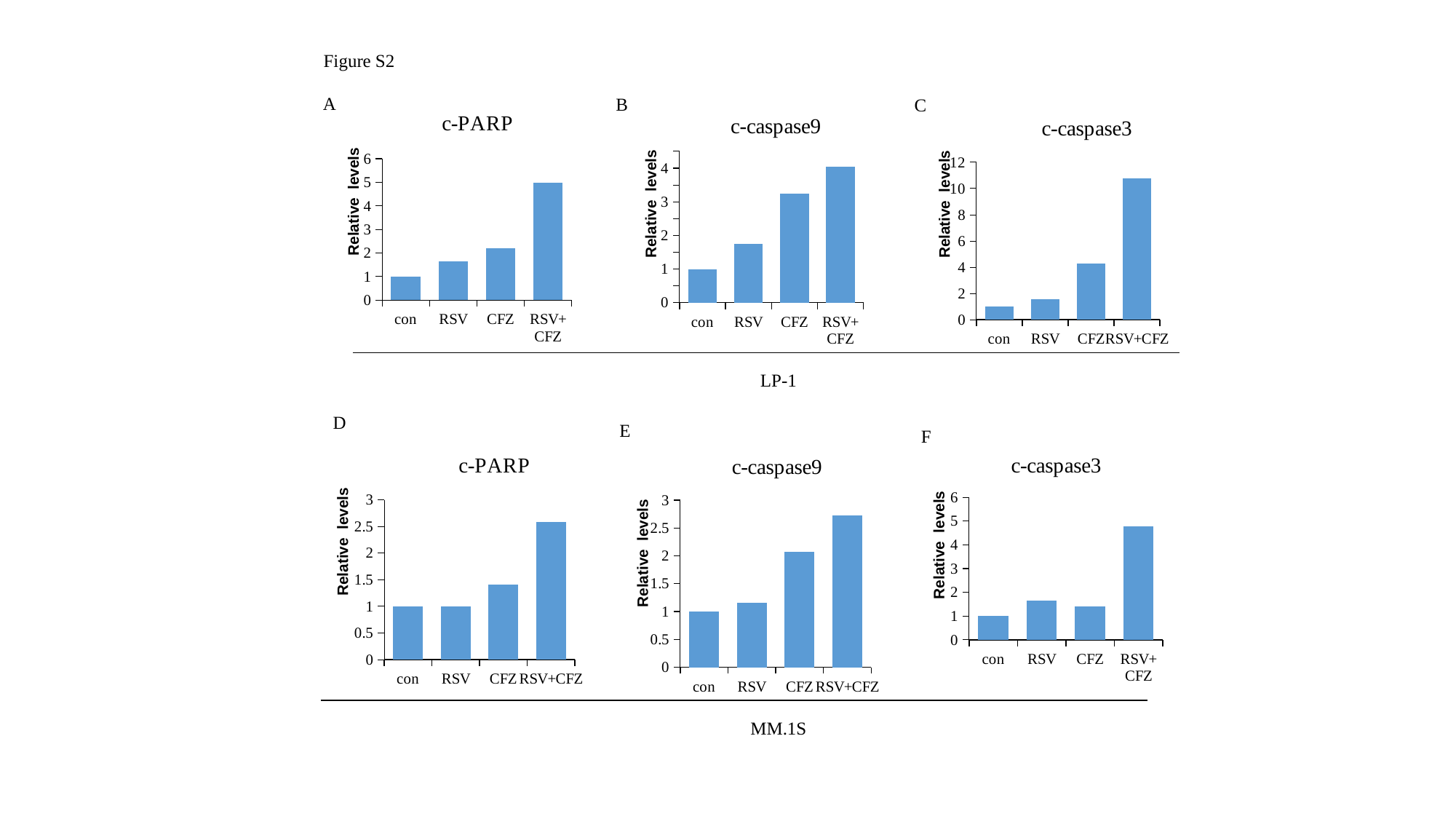

Figure S2
A
B
C
### Chart: c-PARP
| Category | |
|---|---|
| con | 1.0 |
| RSV | 1.6571650306233745 |
| CFZ | 2.205120658196733 |
| RSV+CFZ | 4.9840816797925225 |Relative levels
### Chart: c-caspase9
| Category | |
|---|---|
| con | 1.0 |
| RSV | 1.7514608456047476 |
| CFZ | 3.233427585074324 |
| RSV+CFZ | 4.032782126553846 |Relative levels
### Chart: c-caspase3
| Category | |
|---|---|
| con | 1.0 |
| RSV | 1.5761957058801952 |
| CFZ | 4.268157474204721 |
| RSV+CFZ | 10.761189920676914 |Relative levels
LP-1
D
E
F
### Chart: c-caspase3
| Category | |
|---|---|
| con | 1.0 |
| RSV | 1.6484696165010115 |
| CFZ | 1.4023909383602704 |
| RSV+CFZ | 4.787190224941084 |Relative levels
### Chart: c-caspase9
| Category | |
|---|---|
| con | 1.0 |
| RSV | 1.1587251391021114 |
| CFZ | 2.0776592531432563 |
| RSV+CFZ | 2.724831370949493 |Relative levels
### Chart: c-PARP
| Category | |
|---|---|
| con | 1.0 |
| RSV | 0.9979819840615441 |
| CFZ | 1.4117281714878 |
| RSV+CFZ | 2.5855857342472937 |Relative levels
MM.1S
