# Supplementary material for: Resveratrol Sensitizes Carfilzomib-Induced Apoptosis via Promoting Oxidative Stress in Multiple Myeloma Cells
Source: Front Pharmacol. 2018 May 14;9:334. doi: 10.3389/fphar.2018.00334 (PMC5961230; doi:10.3389/fphar.2018.00334)
Supplement: Supplementary file 4 [file Presentation_3.PPTX]

## Slide 1
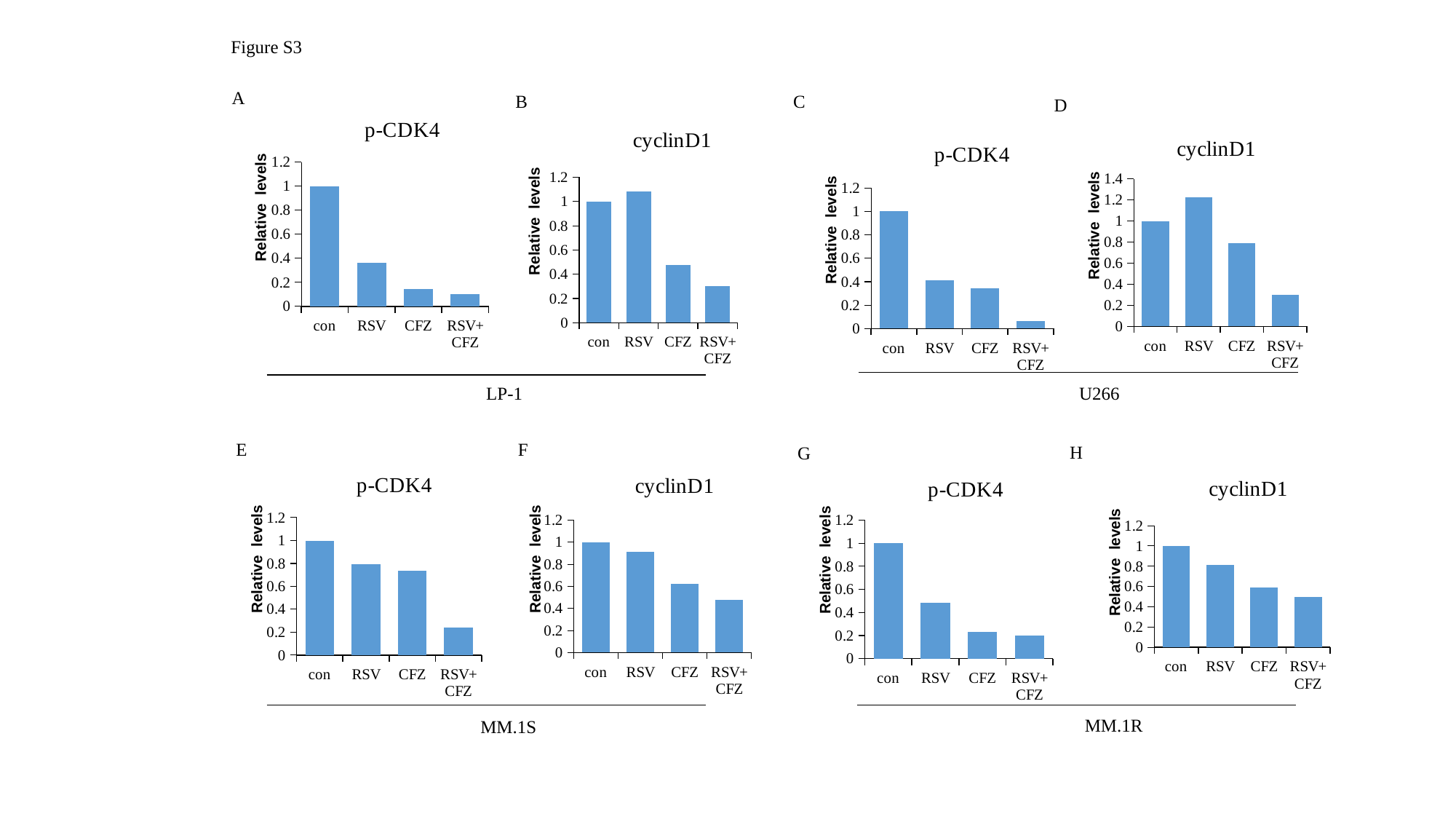

Figure S3
A
C
B
D
### Chart: p-CDK4
| Category | |
|---|---|
| con | 1.0 |
| RSV | 0.3635979285636049 |
| CFZ | 0.1434508569531863 |
| RSV+CFZ | 0.09859825163412562 |Relative levels
### Chart: cyclinD1
| Category | |
|---|---|
| con | 1.0 |
| RSV | 1.084417129600727 |
| CFZ | 0.476188946581005 |
| RSV+CFZ | 0.3 |Relative levels
### Chart: cyclinD1
| Category | |
|---|---|
| con | 1.0 |
| RSV | 1.2217432681816918 |
| CFZ | 0.7930638580581074 |
| RSV+CFZ | 0.29707407315982876 |Relative levels
### Chart: p-CDK4
| Category | |
|---|---|
| con | 1.0 |
| RSV | 0.41067191995407365 |
| CFZ | 0.3436908956852852 |
| RSV+CFZ | 0.06661025025711893 |Relative levels
U266
LP-1
E
F
H
G
### Chart: p-CDK4
| Category | |
|---|---|
| con | 1.0 |
| RSV | 0.7921413883931681 |
| CFZ | 0.7375227794784001 |
| RSV+CFZ | 0.2431314492457362 |Relative levels
### Chart: cyclinD1
| Category | |
|---|---|
| con | 1.0 |
| RSV | 0.9118553313305519 |
| CFZ | 0.6224788408258408 |
| RSV+CFZ | 0.47819962671509647 |Relative levels
### Chart: p-CDK4
| Category | |
|---|---|
| con | 1.0 |
| RSV | 0.48596763962359646 |
| CFZ | 0.23327073793779513 |
| RSV+CFZ | 0.19897051738961563 |Relative levels
### Chart: cyclinD1
| Category | |
|---|---|
| con | 1.0 |
| RSV | 0.8103643639137973 |
| CFZ | 0.5886486204879567 |
| RSV+CFZ | 0.49947878157262743 |Relative levels
MM.1R
MM.1S
