# Supplementary material for: Resveratrol Sensitizes Carfilzomib-Induced Apoptosis via Promoting Oxidative Stress in Multiple Myeloma Cells
Source: Front Pharmacol. 2018 May 14;9:334. doi: 10.3389/fphar.2018.00334 (PMC5961230; doi:10.3389/fphar.2018.00334)
Supplement: Supplementary file 5 [file Presentation_4.PPTX]

## Slide 1
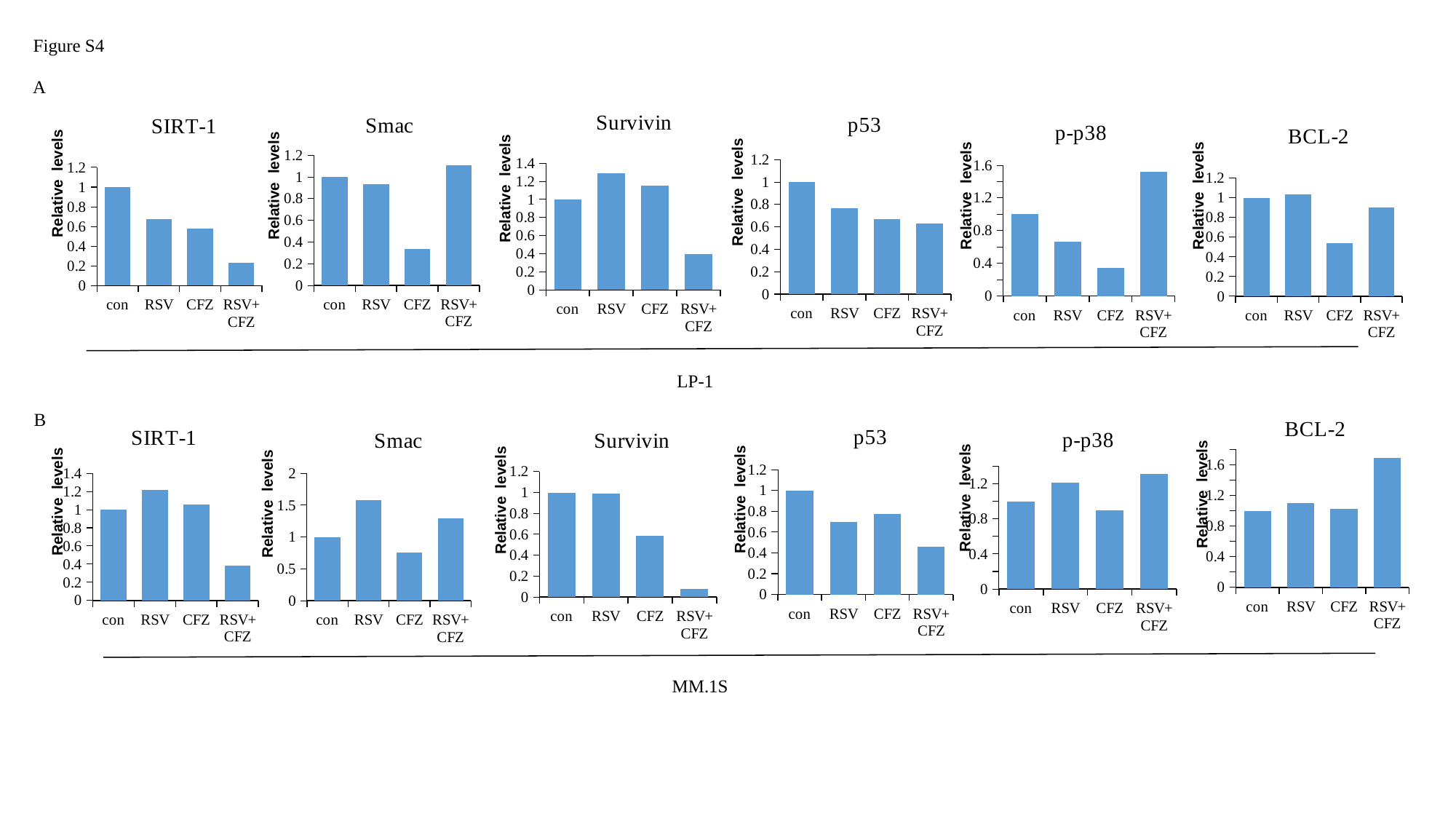

Figure S4
A
### Chart: Smac
| Category | |
|---|---|
| con | 1.0 |
| RSV | 0.9362111811188027 |
| CFZ | 0.3350960828708213 |
| RSV+CFZ | 1.1084923358505694 |Relative levels
### Chart: p53
| Category | |
|---|---|
| con | 1.0 |
| RSV | 0.7654491292729504 |
| CFZ | 0.6666658019709922 |
| RSV+CFZ | 0.632713527108495 |Relative levels
### Chart: Survivin
| Category | |
|---|---|
| con | 1.0 |
| RSV | 1.2897862977757055 |
| CFZ | 1.1489674900621933 |
| RSV+CFZ | 0.3936519837892966 |Relative levels
### Chart: SIRT-1
| Category | |
|---|---|
| con | 1.0 |
| RSV | 0.6771642345993778 |
| CFZ | 0.5809786082241363 |
| RSV+CFZ | 0.23220796073916225 |Relative levels
### Chart: p-p38
| Category | |
|---|---|
| con | 1.0 |
| RSV | 0.6617219597693317 |
| CFZ | 0.3444135464723948 |
| RSV+CFZ | 1.5209487105690065 |Relative levels
### Chart: BCL-2
| Category | |
|---|---|
| con | 1.0 |
| RSV | 1.0334781768779868 |
| CFZ | 0.5408204087546684 |
| RSV+CFZ | 0.900310075307106 |Relative levels
LP-1
### Chart: BCL-2
| Category | |
|---|---|
| con | 1.0 |
| RSV | 1.099775046366762 |
| CFZ | 1.021540375414805 |
| RSV+CFZ | 1.692888886740769 |Relative levels
B
### Chart: p53
| Category | |
|---|---|
| con | 1.0 |
| RSV | 0.6986470013036329 |
| CFZ | 0.7735687581739964 |
| RSV+CFZ | 0.4600559139034029 |Relative levels
### Chart: Survivin
| Category | |
|---|---|
| con | 1.0 |
| RSV | 0.9903184598958057 |
| CFZ | 0.5887198998175964 |
| RSV+CFZ | 0.07451563426507915 |Relative levels
### Chart: SIRT-1
| Category | |
|---|---|
| con | 1.0 |
| RSV | 1.2181862382680786 |
| CFZ | 1.059188154369694 |
| RSV+CFZ | 0.38128211805024526 |Relative levels
### Chart: Smac
| Category | |
|---|---|
| con | 1.0 |
| RSV | 1.578158725424601 |
| CFZ | 0.7529632185446093 |
| RSV+CFZ | 1.2945222823642004 |Relative levels
### Chart: p-p38
| Category | |
|---|---|
| con | 1.0 |
| RSV | 1.213033983753573 |
| CFZ | 0.9017277176719606 |
| RSV+CFZ | 1.3099970030264012 |Relative levels
MM.1S
