# Supplementary material for: Resveratrol Sensitizes Carfilzomib-Induced Apoptosis via Promoting Oxidative Stress in Multiple Myeloma Cells
Source: Front Pharmacol. 2018 May 14;9:334. doi: 10.3389/fphar.2018.00334 (PMC5961230; doi:10.3389/fphar.2018.00334)
Supplement: Supplementary file 6 [file Presentation_5.PPTX]

## Slide 1
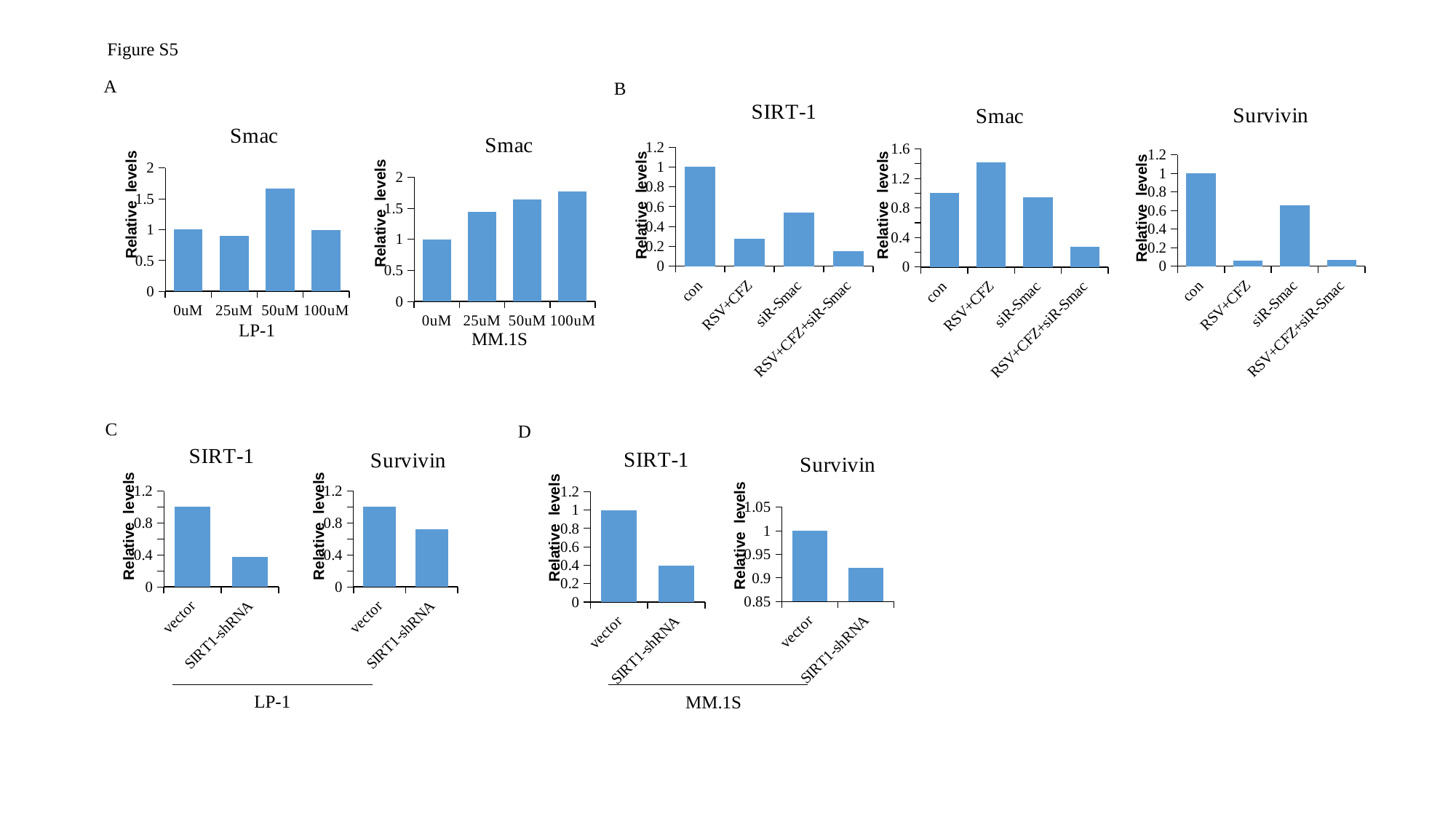

Figure S5
A
B
### Chart: SIRT-1
| Category | |
|---|---|
| con | 1.0 |
| RSV+CFZ | 0.27319632445298897 |
| siR-Smac | 0.5402572800808434 |
| RSV+CFZ+siR-Smac | 0.1535623391311111 |Relative levels
### Chart: Smac
| Category | |
|---|---|
| con | 1.0 |
| RSV+CFZ | 1.4133247516905432 |
| siR-Smac | 0.9454897874684807 |
| RSV+CFZ+siR-Smac | 0.2761664815885654 |Relative levels
### Chart: Survivin
| Category | |
|---|---|
| con | 1.0 |
| RSV+CFZ | 0.0640427059669174 |
| siR-Smac | 0.6565237566834652 |
| RSV+CFZ+siR-Smac | 0.0706701212340549 |Relative levels
### Chart: Smac
| Category | |
|---|---|
| 0uM | 1.0 |
| 25uM | 0.8951178881882393 |
| 50uM | 1.6669862334579126 |
| 100uM | 0.9969492826301196 |Relative levels
LP-1
### Chart: Smac
| Category | |
|---|---|
| 0uM | 1.0 |
| 25uM | 1.4397431324282526 |
| 50uM | 1.6395506832782039 |
| 100uM | 1.7665890498677475 |Relative levels
MM.1S
C
D
### Chart: SIRT-1
| Category | |
|---|---|
| vector | 1.0 |
| SIRT1-shRNA | 0.37355653667831595 |Relative levels
### Chart: Survivin
| Category | |
|---|---|
| vector | 1.0 |
| SIRT1-shRNA | 0.7210237514541764 |Relative levels
### Chart: SIRT-1
| Category | |
|---|---|
| vector | 1.0 |
| SIRT1-shRNA | 0.39776735745323877 |Relative levels
### Chart: Survivin
| Category | |
|---|---|
| vector | 1.0 |
| SIRT1-shRNA | 0.9215397165697861 |Relative levels
LP-1
MM.1S
