# Supplementary material for: Resveratrol Sensitizes Carfilzomib-Induced Apoptosis via Promoting Oxidative Stress in Multiple Myeloma Cells
Source: Front Pharmacol. 2018 May 14;9:334. doi: 10.3389/fphar.2018.00334 (PMC5961230; doi:10.3389/fphar.2018.00334)
Supplement: Supplementary file 7 [file Presentation_6.PPTX]

## Slide 1
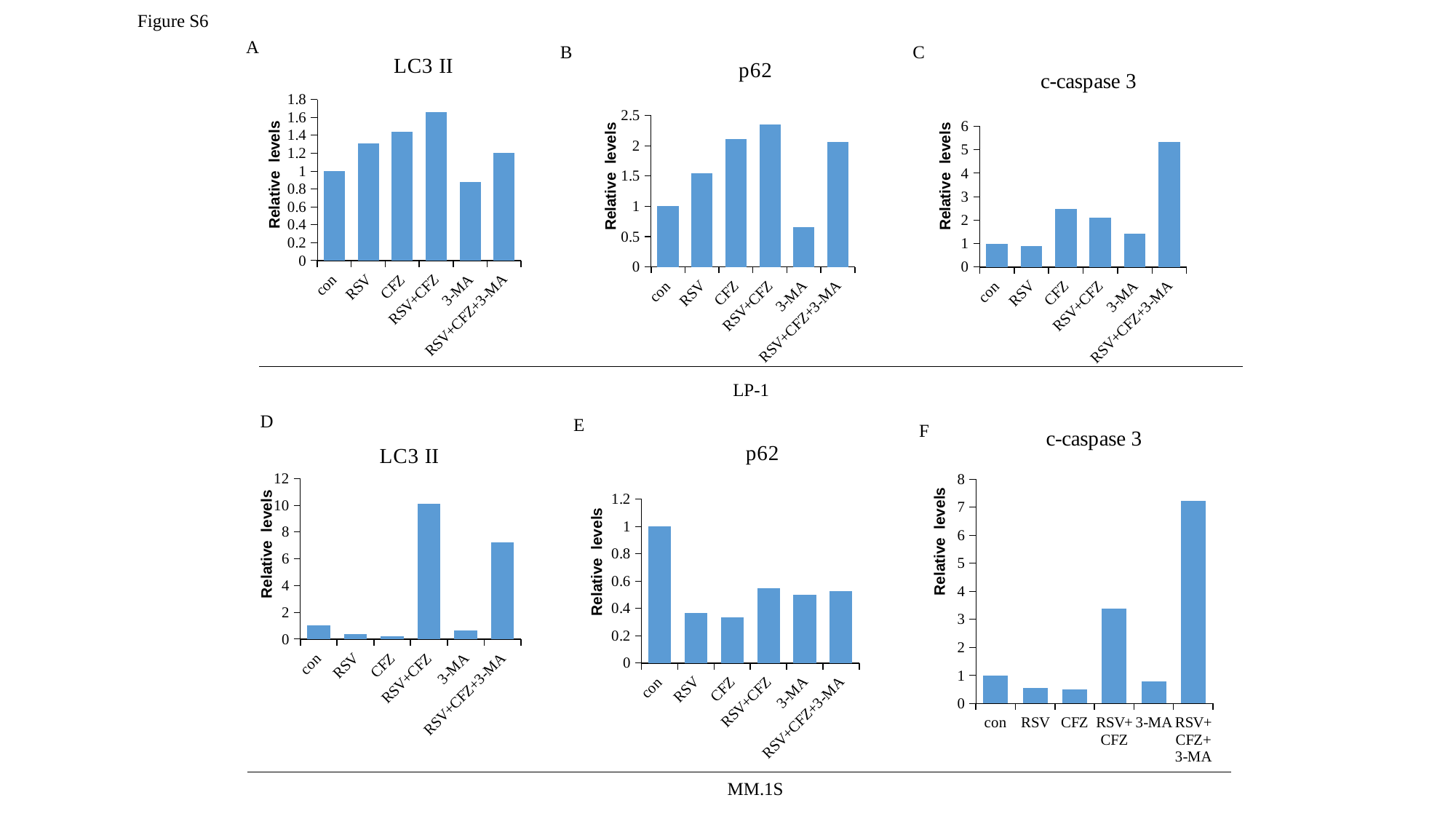

Figure S6
A
C
B
### Chart: LC3 II
| Category | |
|---|---|
| con | 1.0 |
| RSV | 1.313299820853031 |
| CFZ | 1.4366255286073488 |
| RSV+CFZ | 1.6601698192847472 |
| 3-MA | 0.8781675283814222 |
| RSV+CFZ+3-MA | 1.2029121947526862 |
### Chart: p62
| Category | |
|---|---|
| con | 1.0 |
| RSV | 1.5444246165458975 |
| CFZ | 2.1153225708086416 |
| RSV+CFZ | 2.3491957391874045 |
| 3-MA | 0.6565470224222354 |
| RSV+CFZ+3-MA | 2.065858282902069 |
### Chart: c-caspase 3
| Category | |
|---|---|
| con | 1.0 |
| RSV | 0.908765456364806 |
| CFZ | 2.485836739512419 |
| RSV+CFZ | 2.097043812056357 |
| 3-MA | 1.4236061639359843 |
| RSV+CFZ+3-MA | 5.340553979613586 |Relative levels
Relative levels
Relative levels
LP-1
D
E
F
### Chart: c-caspase 3
| Category | |
|---|---|
| con | 1.0 |
| RSV | 0.5472064359911327 |
| CFZ | 0.514322324761983 |
| RSV+CFZ | 3.3933917552859687 |
| 3-MA | 0.8027113477897305 |
| RSV+CFZ+3-MA | 7.222797180203319 |Relative levels
### Chart: LC3 II
| Category | |
|---|---|
| con | 1.0 |
| RSV | 0.3707015028782034 |
| CFZ | 0.22185049434180518 |
| RSV+CFZ | 10.127947415945776 |
| 3-MA | 0.6659738718672723 |
| RSV+CFZ+3-MA | 7.231452154098521 |Relative levels
### Chart: p62
| Category | |
|---|---|
| con | 1.0 |
| RSV | 0.3670286719957678 |
| CFZ | 0.3374021084636809 |
| RSV+CFZ | 0.5506919078998563 |
| 3-MA | 0.5011668355923773 |
| RSV+CFZ+3-MA | 0.5284267418306886 |Relative levels
MM.1S
